# Supplementary material for: The association between racism and psychosis: An umbrella review
Source: PLOS Ment Health. 2025 Sep 24;2(9):e0000401. doi: 10.1371/journal.pmen.0000401 (PMC12798482; doi:10.1371/journal.pmen.0000401)
Supplement: S1 Text — (DOCX) [file pmen.0000401.s002.docx]

## S1 Text. Umbrella Review Search Strategy.

**Search strategy**

We followed the Preferred Reporting Items for Systematic Reviews and Meta-Analyses (PRISMA) guidelines. An initial search of the following databases was performed on 8^th^ March 2023: Medline via Ovid, Embase via Ovid, PsycINFO via Ovid and ProQuest Central. The search strategy included terms relating to two domains, namely racism and psychosis. The Medline and Embase searches were linked to adapted versions (inclusion of lines 1–6 and 1–3, respectively) of the SIGN filters for Systematic Reviews (https://sites.google.com/a/york.ac.uk/issg-search-filters-resource/home/systematic-reviews?pli=1). The adapted Medline filter was used for the PsycInfo and ProQuest Central searches.

This initial search had no restriction on date of publication but with language restricted to English only. The search was re-run without any date or language restrictions on 19^th^ July 2024. Non-English language reviews from the initial search were retrospectively re-identified and screened.

A separate search to find additional missing reviews was performed in Google Scholar (https://scholar.google.com/) on 8^th^ March 2023 using the following search strings:

- "racism" AND "psychosis" AND "systematic review"
- "racism" AND "psychosis" AND "meta-analysis"
- "racism" AND "schizophrenia" AND "systematic review"
- "racism" AND "schizophrenia" AND " meta-analysis"
- "racial discrimination" AND "psychosis" AND "systematic review"
- "racial discrimination" AND "psychosis" AND "meta-analysis"
- "racial discrimination" AND "schizophrenia" AND "systematic review"
- "racial discrimination" AND "schizophrenia" AND " meta-analysis"

We selected the first 50 results for each search string outlined above (400 results in total) which were then screened alongside the records from the four databases. We believed this would provide sufficient additional searching of the Google Scholar database on top of our comprehensive of our four aforementioned datasets and the forward and backward citation searching.

We also performed forward- and backward-citation searching from the included reviews on 16^th^ November 2023 and 17^th^ November 2023, respectively, to identify any other relevant reviews. As no additional reviews were identified when the search was re-run on 19^th^ July 2024, no forward- or backward-citation searching was performed. We conducted forward searching via the Web of Science citation feature, employing a ‘review only’ filter where there were over 50 citations of the review.

The search was restricted to systematic reviews and meta-analyses published in peer-reviewed academic journals.

**Searches**

Medline (via Ovid)

1. raci*
2. ethnic* bias*
3. (perceiv* adj3 discriminat*)
4. (ethnic* adj4 discriminat*)
5. (ethnic* adj5 (inequit* or disparit* or inequalit*))
6. ((prejudice* or disadvantage* or injustice* or inequalit* or discriminat* or bias* or disparit* or inequit* or stereotyp*) and ("black, asian and minority ethnic*" or "BAME" or "black and minority ethic*" or "BME" or "black, indigenous, and people of color" or "black, indigenous, and people of colour" or "BIPOC" or "people of color" or "people of colour" or "non-white*" or "black*" or "asian*" or "latin*" or "hispanic*" or "indigenous" or "mixed-heritage" or "mixed-race" or "multi-ethnic" or "dual-heritage" or "multi-heritage" or "biracial" or "white*" or "african*" or "african-american*" or "afro-caribbean*" or "caribbean*" or "european*" or "minorit* group*" or "global majority" or "global south"))
7. Racism/
8. Systemic Racism/
9. Perceived Discrimination/
10. Prejudice/ or Social Discrimination/
11. Racial Groups/
12. Ethnicity/
13. "Ethnic and Racial Minorities"/
14. Minority Groups/
15. Health Status Disparities/
16. 1 or 2 or 3 or 4 or 5 or 6 or 7 or 8 or 9 or 10 or 11 or 12 or 13 or 14 or 15
17. psychos?s*
18. psychotic*
19. schizo*
20. severe mental illness*
21. severe mental disorder*
22. severe mental health condition*
23. manic* depress*
24. paranoi*
25. hallucinat*
26. delusion*
27. psychotic disorders/
28. affective disorders, psychotic/
29. Psychoses, Alcoholic/
30. Psychoses, Substance-Induced/
31. Capgras Syndrome/
32. schizophrenia/
33. schizophrenia, catatonic/
34. schizophrenia, childhood/
35. schizophrenia, disorganized/
36. schizophrenia, paranoid/
37. schizophrenia, treatment-resistant/
38. schizoid personality disorder/
39. schizotypal personality disorder/
40. "schizophrenia spectrum and other psychotic disorders"/
41. bipolar disorder/
42. paranoid behavior/
43. paranoid disorders/
44. hallucinations/
45. delusions/
46. 17 or 18 or 19 or 20 or 21 or 22 or 23 or 24 or 25 or 26 or 27 or 28 or 29 or 30 or 31 or 32 or 33 or 34 or 35 or 36 or 37 or 38 or 39 or 40 or 41 or 42 or 43 or 44 or 45
47. narrative review*.tw,kf.
48. "systematic review"/
49. "Review"/
50. "Review Literature as Topic"/
51. Meta-Analysis as Topic/
52. meta analy$.tw.
53. metaanaly$.tw.
54. Meta-Analysis/
55. (systematic adj (review$1 or overview$1)).tw.
56. exp Review Literature as Topic/
57. 47 or 48 or 49 or 50 or 51 or 52 or 53 or 54 or 55 or 56
58. 16 and 46 and 57

Lines 51–56 are linked to an adapted version of the SIGN Medline Ovid filter (lines 1–6) for Systematic Reviews (https://sites.google.com/a/york.ac.uk/issg-search-filters-resource/home/systematic-reviews?pli=1).

Embase (via Ovid)

1. raci*
2. ethnic* bias*
3. (perceiv* adj3 discriminat*)
4. (ethnic* adj4 discriminat*)
5. (ethnic* adj5 (inequit* or disparit* or inequalit*))
6. ((prejudice* or disadvantage* or injustice* or inequalit* or discriminat* or bias* or disparit* or inequit* or stereotyp*) and ("black, asian and minority ethnic*" or "BAME" or "black and minority ethic*" or "BME" or "black, indigenous, and people of color" or "black, indigenous, and people of colour" or "BIPOC" or "people of color" or "people of colour" or "non-white*" or "black*" or "asian*" or "latin*" or "hispanic*" or "indigenous" or "mixed-heritage" or "mixed-race" or "multi-ethnic" or "dual-heritage" or "multi-heritage" or "biracial" or "white*" or "african*" or "african-american*" or "afro-caribbean*" or "caribbean*" or "european*" or "minorit* group*" or "global majority" or "global south"))
7. racism/
8. structural racism/
9. perceived discrimination/
10. social discrimination/ or prejudice/
11. racial disparity/
12. race/
13. ethnicity/
14. ethnic group/
15. ethnic identity/
16. minority group/
17. health disparity/
18. 1 or 2 or 3 or 4 or 5 or 6 or 7 or 8 or 9 or 10 or 11 or 12 or 13 or 14 or 15 or 16 or 17
19. psychos?s*
20. psychotic*
21. schizo*
22. severe mental illness*
23. severe mental disorder*
24. severe mental health condition*
25. manic* depress*
26. paranoi*
27. hallucinat*
28. delusion*
29. psychosis/
30. acute psychosis/
31. affective psychosis/
32. alcohol psychosis/
33. brief psychotic disorder/
34. childhood psychosis/
35. depressive psychosis/
36. drug induced psychosis/
37. endogenous psychosis/
38. intensive care psychosis/
39. manic psychosis/
40. menstrual psychosis/
41. paranoid psychosis/
42. puerperal psychosis/
43. Capgras Syndrome/
44. schizophrenia/
45. catatonic schizophrenia/
46. hebephrenia/
47. latent schizophrenia/
48. paranoid schizophrenia/
49. residual schizophrenia/
50. simple schizophrenia/
51. treatment-resistant schizophrenia/
52. schizoaffective psychosis/
53. schizoidism/
54. schizotypal personality disorder/
55. schizophrenia spectrum disorder/
56. schizophreniform disorder/
57. bipolar disorder/
58. paranoia/
59. hallucination/
60. delusion/
61. delusional disorder/
62. 19 or 20 or 21 or 22 or 23 or 24 or 25 or 26 or 27 or 28 or 29 or 30 or 31 or 32 or 33 or 34 or 35 or 36 or 37 or 38 or 39 or 40 or 41 or 42 or 43 or 44 or 45 or 46 or 47 or 48 or 49 or 50 or 51 or 52 or 53 or 54 or 55 or 56 or 57 or 58 or 59 or 60 or 61
63. narrative review*.tw,kf.
64. "systematic review"/
65. "systematic review (topic)"/
66. "review"/
67. meta analysis/
68. "meta analysis (topic)"/
69. exp Meta Analysis/
70. ((meta adj analy$) or metaanalys$).tw.
71. (systematic adj (review$1 or overview$1)).tw.
72. 63 or 64 or 65 or 66 or 67 or 68 or 69 or 70 or 71
73. 18 and 62 and 72

Lines 69–71 are linked to an adapted version of the SIGN Embase Ovid filter (lines 1–3) for Systematic Reviews (https://sites.google.com/a/york.ac.uk/issg-search-filters-resource/home/systematic-reviews?pli=1).

PsycInfo (via Ovid)

1. raci*
2. ethnic* bias*
3. (perceiv* adj3 discriminat*)
4. (ethnic* adj4 discriminat*)
5. (ethnic* adj5 (inequit* or disparit* or inequalit*))
6. ((prejudice* or disadvantage* or injustice* or inequalit* or discriminat* or bias* or disparit* or inequit* or stereotyp*) and ("black, asian and minority ethnic*" or "BAME" or "black and minority ethic*" or "BME" or "black, indigenous, and people of color" or "black, indigenous, and people of colour" or "BIPOC" or "people of color" or "people of colour" or "non-white*" or "black*" or "asian*" or "latin*" or "hispanic*" or "indigenous" or "mixed-heritage" or "mixed-race" or "multi-ethnic" or "dual-heritage" or "multi-heritage" or "biracial" or "white*" or "african*" or "african-american*" or "afro-caribbean*" or "caribbean*" or "european*" or "minorit* group*" or "global majority" or "global south"))
7. Racism/
8. Social Discrimination/ or Prejudice/
9. "Race and Ethnic Discrimination"/
10. "Racial and Ethnic Differences"/
11. Racial Bias/
12. Racial Disparities/
13. Ethnic Identity/
14. "Racial and Ethnic Groups"/
15. Minority Groups/
16. Health Disparities/
17. 1 or 2 or 3 or 4 or 5 or 6 or 7 or 8 or 9 or 10 or 11 or 12 or 13 or 14 or 15 or 16
18. psychos?s*
19. psychotic*
20. schizo*
21. severe mental illness*
22. severe mental disorder*
23. severe mental health condition*
24. manic* depress*
25. paranoi*
26. hallucinat*
27. delusion*
28. Psychosis/
29. acute psychosis/
30. Affective Psychosis/
31. Schizoaffective Disorder/
32. Alcoholic Psychosis/
33. Childhood Psychosis/
34. Chronic Psychosis/
35. "Paranoia (Psychosis)"/
36. Postpartum Psychosis/
37. Reactive Psychosis/
38. Capgras Syndrome/
39. Schizophrenia/
40. Acute Schizophrenia/
41. Catatonic Schizophrenia/
42. Childhood Schizophrenia/
43. "Schizophrenia (Disorganized Type)"/
44. "Fragmentation (Schizophrenia)"/
45. Paranoid Schizophrenia/
46. Process Schizophrenia/
47. Undifferentiated Schizophrenia/
48. Schizoid Personality Disorder/
49. Schizotypal Personality Disorder/
50. Schizotypy/
51. Schizophreniform Disorder/
52. Bipolar Disorder/
53. Paranoia/
54. Hallucinations/
55. Delusions/
56. Psychiatric Symptoms/
57. 18 or 19 or 20 or 21 or 22 or 23 or 24 or 25 or 26 or 27 or 28 or 29 or 30 or 31 or 32 or 33 or 34 or 35 or 36 or 37 or 38 or 39 or 40 or 41 or 42 or 43 or 44 or 45 or 46 or 47 or 48 or 49 or 50 or 51 or 52 or 53 or 54 or 55 or 56
58. narrative review*.tw,id.
59. "Systematic Review"/
60. "Literature Review"/
61. meta analy$.tw.
62. metaanaly$.tw.
63. Meta Analysis/
64. (systematic adj (review$1 or overview$1)).tw.
65. 58 or 59 or 60 or 61 or 62 or 63 or 64
66. 17 and 57 and 65

Lines 61–64 are linked to an adapted version of the SIGN Medline Ovid filter (lines 1–6) for Systematic Reviews (https://sites.google.com/a/york.ac.uk/issg-search-filters-resource/home/systematic-reviews?pli=1).

Proquest Central

1. raci*
2. "ethnic* bias*"
3. perceiv* NEAR/2 discriminat*
4. ethnic* NEAR/3 discriminat*
5. ethnic* NEAR/4 (inequit* OR disparit* OR inequalit*)
6. (prejudice* or disadvantage* or injustice* or inequalit* or discriminat* or bias* or disparit* or inequit* or stereotyp*) and ("black, asian and minority ethnic*" or "BAME" or "black and minority ethic*" or "BME" or "black, indigenous, and people of color" or "black, indigenous, and people of colour" or "BIPOC" or "people of color" or "people of colour" or "non-white*" or "black*" or "asian*" or "latin*" or "hispanic*" or "indigenous" or "mixed-heritage" or "mixed-race" or "multi-ethnic" or "dual-heritage" or "multi-heritage" or "biracial" or "white*" or "african*" or "african-american*" or "afro-caribbean*" or "caribbean*" or "european*" or "minorit* group*" or "global majority" or "global south")
7. MAINSUBJECT.EXACT("Racism")
8. MAINSUBJECT.EXACT("Racial discrimination")
9. MAINSUBJECT.EXACT("Racial harassment")
10. MAINSUBJECT.EXACT("Racial profiling")
11. MAINSUBJECT.EXACT("Bias")
12. MAINSUBJECT.EXACT("Prejudice")
13. MAINSUBJECT.EXACT("Race relations")
14. MAINSUBJECT.EXACT("Race")
15. MAINSUBJECT.EXACT("Ethnicity")
16. MAINSUBJECT.EXACT("Racial identity")
17. MAINSUBJECT.EXACT("Minority & ethnic groups")
18. MAINSUBJECT.EXACT("Health disparities")
19. 1 or 2 or 3 or 4 or 5 or 6 or 7 or 8 or 9 or 10 or 11 or 12 or 13 or 14 or 15 or 16 or 17 or 18
20. psychos?s*
21. psychotic*
22. schizo*
23. "severe mental illness*"
24. "severe mental disorder*"
25. "severe mental health condition*"
26. "manic* depress*"
27. paranoi*
28. hallucinat*
29. delusion*
30. MAINSUBJECT.EXACT("Psychosis")
31. MAINSUBJECT.EXACT("Schizophrenia")
32. MAINSUBJECT.EXACT("Bipolar disorder")
33. MAINSUBJECT.EXACT("Paranoia")
34. MAINSUBJECT.EXACT("Hallucinations")
35. 20 or 21 or 22 or 23 or 24 or 25 or 26 or 27 or 28 or 29 or 30 or 31 or 32 or 33 or 34
36. "narrative review*"
37. "meta analy*"
38. "metaanaly*"
39. systematic NEAR/0 (review* or overview*)
40. MAINSUBJECT.EXACT("Meta-analysis")
41. MAINSUBJECT.EXACT("Literature reviews")
42. MAINSUBJECT.EXACT("Systematic review")
43. 36 or 37 or 38 or 39 or 40 or 41 or 42
44. 19 and 35 and 43

Lines 37–41 are linked to an adapted version of the SIGN Medline Ovid filter (lines 1–6) for Systematic Reviews (https://sites.google.com/a/york.ac.uk/issg-search-filters-resource/home/systematic-reviews?pli=1).

Proquest Central was interrogated for each terms using the search parameter ‘Anywhere but full text – NOFT’.
