# Supplementary material for: The association between racism and psychosis: An umbrella review
Source: PLOS Ment Health. 2025 Sep 24;2(9):e0000401. doi: 10.1371/journal.pmen.0000401 (PMC12798482; doi:10.1371/journal.pmen.0000401)
Supplement: S2 Text — (DOCX) [file pmen.0000401.s003.docx]

## S2 Text. Additional Results: Mediators/Moderators of the association between racial/ethnic discrimination and psychosis outcomes in non-clinical samples.

Several reviews reported findings on the potential mediating and moderating effects of social support, ethnic density, race-based rejection sensitivity, ethnic identity and self-esteem on the association between racial/ethnic discrimination and non-clinical psychotic outcomes. Bardol et al. [[1](#_ENREF_1)] reported two studies conducted in The Netherlands which investigated potential moderators of the association between racial/ethnic discrimination and psychosis symptoms. The first one identified that social support significantly moderated the association in a sample of 267 participants (R^2^ change 0.021, p = 0.013) [[2](#_ENREF_2)], however, this was contrasted by a study reported by Bardol et al. [[1](#_ENREF_1)], Paradies et al. [[3](#_ENREF_3)] and Pearce et al. [[4](#_ENREF_4)] which found that social support did not moderate the association between racial/ethnic discrimination and psychosis symptoms in a UK sample of 4281 participants [[5](#_ENREF_5)]. The second study reported only by Bardol et al. [[1](#_ENREF_1)] failed to identify a buffering effect of ethnic density on the association in a sample of 1186 participants [[6](#_ENREF_6)]. Similarly, a UK study reported by Bardol et al. [[1](#_ENREF_1)], Paradies et al. [[3](#_ENREF_3)] and Pearce et al. [[4](#_ENREF_4)] also did not find a significant moderating effect of high ethnic density on the relationship [[7](#_ENREF_7)]. However, another study did find ethnic density effects - an increased likelihood of people from racially/ethnically marginalised backgrounds reporting psychotic experiences when own-group ethnic density decreased by 10% and increased reports of racial/ethnic discrimination and lower social support from racially/ethnically marginalised people in low own-group ethnic density areas [[8](#_ENREF_8)].

Two studies [[9](#_ENREF_9), [10](#_ENREF_10)] reported by both Bardol et al. [[1](#_ENREF_1)] and Pearce et al. [[4](#_ENREF_4)], investigated the relationship between lifetime perceived racial/ethnic discrimination and attenuated positive psychosis symptoms, and distress associated with these symptoms. They

found evidence of a small, partial mediating effect on the relationship of the participant’s sensitivity to race-based rejection [[9](#_ENREF_9)] as well as a significant buffering effect of strong ethnic identity (b = −0.45, S.E. = 0.20, p < 0.05) [[10](#_ENREF_10)].

A final study [[11](#_ENREF_11)], reported by both Bardol et al. [[1](#_ENREF_1)] and Pearce et al. [[4](#_ENREF_4)], found that collective self-esteem was a moderator of the relationship between lifetime perceived racial/ethnic discrimination and paranoia, with high collective self-esteem leading to the association between the two no longer being significant.

References

1. Bardol O, Grot S, Oh H, Poulet E, Zeroug-Vial H, Brunelin J, et al. Perceived ethnic discrimination as a risk factor for psychotic symptoms: a systematic review and meta-analysis. Psychological medicine. 2020;50(7):1077-89. doi: <https://dx.doi.org/10.1017/S003329172000094X>.

2. van de Beek MH, van der Krieke L, Schoevers RA, Veling W. Social exclusion and psychopathology in an online cohort of Moroccan-Dutch migrants: Results of the MEDINA-study. PLoS One. 2017;12(7). doi: <https://doi.org/10.1371/journal.pone.0179827>. PubMed PMID: 1917696424.

3. Paradies Y, Ben J, Denson N, Elias A, Priest N, Pieterse A, et al. Racism as a determinant of health: a systematic review and meta-analysis. PloS one. 2015;10(9):e0138511.

4. Pearce J, Rafiq S, Simpson J, Varese F. Perceived discrimination and psychosis: a systematic review of the literature. Social psychiatry and psychiatric epidemiology. 2019;54(9):1023-44. doi: <https://dx.doi.org/10.1007/s00127-019-01729-3>.

5. Chakraborty AT, McKenzie KJ, Hajat S, Stansfeld SA. Racism, mental illness and social support in the UK. Soc Psychiatry Psychiatr Epidemiol. 2010;45(12):1115-24. Epub 20091022. doi: 10.1007/s00127-009-0156-8. PubMed PMID: 19847373.

6. El Bouhaddani S, van Domburgh L, Schaefer B, Doreleijers TAH, Veling W. Psychotic experiences among ethnic majority and minority adolescents and the role of discrimination and ethnic identity. Soc Psychiatry Psychiatr Epidemiol. 2019;54(3):343-53. Epub 20190114. doi: 10.1007/s00127-019-01658-1. PubMed PMID: 30643926.

7. Bécares L, Nazroo J, Stafford M. The buffering effects of ethnic density on experienced racism and health. Health Place. 2009;15(3):670-8. Epub 20081118. doi: 10.1016/j.healthplace.2008.10.008. PubMed PMID: 19117792.

8. Das-Munshi J, Bécares L, Boydell JE, Dewey ME, Morgan C, Stansfeld SA, et al. Ethnic density as a buffer for psychotic experiences: findings from a national survey (EMPIRIC). Br J Psychiatry. 2012;201(4):282-90. Epub 20120726. doi: 10.1192/bjp.bp.111.102376. PubMed PMID: 22844021; PubMed Central PMCID: PMCPMC3461446.

9. Anglin DM, Greenspoon M, Lighty Q, Ellman LM. Race-based rejection sensitivity partially accounts for the relationship between racial discrimination and distressing attenuated positive psychotic symptoms. Early Interv Psychiatry. 2016;10(5):411-8. Epub 20140918. doi: 10.1111/eip.12184. PubMed PMID: 25234291.

10. Anglin DM, Lui F, Espinosa A, Tikhonov A, Ellman L. Ethnic identity, racial discrimination and attenuated psychotic symptoms in an urban population of emerging adults. Early Interv Psychiatry. 2018;12(3):380-90. Epub 20160128. doi: 10.1111/eip.12314. PubMed PMID: 26818635.

11. Kong DT. Ethnic minorities' paranoia and self-preservative work behaviors in response to perceived ethnic discrimination, with collective self-esteem as a buffer. J Occup Health Psychol. 2016;21(3):334-51. Epub 20151214. doi: 10.1037/ocp0000013. PubMed PMID: 26652266.
