# Supplementary material for: The association between racism and psychosis: An umbrella review
Source: PLOS Ment Health. 2025 Sep 24;2(9):e0000401. doi: 10.1371/journal.pmen.0000401 (PMC12798482; doi:10.1371/journal.pmen.0000401)
Supplement: S3 Text — (DOCX) [file pmen.0000401.s004.docx]

## S3 Text. Additional Results: Results by racial/ethnic background.

Several reviews reported studies or conducted analyses into the effect of racial/ethnic background on the association with mixed findings. Bardol et al. [[1](#_ENREF_1)] investigated whether perceived ethnic discrimination had similar effects on psychotic experiences/symptoms in different ethnic groups. It found evidence of such associations in four of the five ethnic groups studied (Bangladeshi, Black Caribbean, Indian and Pakistani, but not Irish) with low heterogeneity (I^2^ = 0, Q(13) = 12.09, p = 0.52). Additionally, they found no statistically significant variation in the strength of these associations between ethnic groups (Q = 2.22, df(Q) = 4, p = 0.695). One study included in this review [[2](#_ENREF_2)] identified statistically significant relationships between verbal discrimination and psychotic symptoms in several ethnic groups, including people from Black Caribbean (OR 3.35, 95% CI 1.79, 6.26), Bangladeshi (OR 5.46, 95% CI 1.79, 6.26) and Pakistani ethnic groups (OR 2.65, 95% CI 1.26, 5.55). No statistically significant association was observed for the Indian group (OR 2.27, 95% CI 0.85, 6.06). This study also observed a relationship between job refusal and psychotic symptoms for the Pakistani group (OR 2.26, 95% CI 1.08, 4.75).

A Norwegian primary study conducted in clinical a population [[3](#_ENREF_3)], reported by Bardol et al. [[1](#_ENREF_1)], Paradies et al. [[4](#_ENREF_4)] and Pearce et al. [[5](#_ENREF_5)], found that people from African American backgrounds reported higher rates of perceived racial/ethnic discrimination and had the most severe positive symptoms of the ethnic groups investigated (t = 2.472, df = 88, p < 0.015). The study also found that perceived racial/ethnic discrimination partially mediated the relationship between African immigrant status and symptom severity. Similarly, Bécares et al. [[6](#_ENREF_6)], from Bardol et al. [[1](#_ENREF_1)], Paradies et al. [[4](#_ENREF_4)] and Pearce et al. [[5](#_ENREF_5)], found the greatest effect of racial/ethnic discrimination on psychotic experiences in people from Indian (adjusted OR 4.15, p < 0.001) and Caribbean (adjusted OR 3.47, p < 0.001) backgrounds.

Karlsen et al. [[7](#_ENREF_7)], reported by Bardol et al. [[1](#_ENREF_1)], de Freitas et al. [[8](#_ENREF_8)], Pearce et al. [[5](#_ENREF_5)] and Williams and Mohammed [[9](#_ENREF_9)], found that people from Bangladeshi backgrounds had the greatest psychosis risk (OR 7.83, 95% CI 2.00, 30.61), with lower risk seen in participants from by Caribbean (OR 3.45, 95% CI 1.73, 6.90) and Pakistani (OR 3.36, 95% 1.58, 7.18) backgrounds. Additionally, while the study did not find a significant association between perceived work-related racial/religious discrimination and psychotic experiences across all racially/ethnically marginalised groups, an increased risk was seen in Caribbean participants when they perceived their employer to be racist (OR 2.34, 95% CI 1.28, 4.28).

References

1. Bardol O, Grot S, Oh H, Poulet E, Zeroug-Vial H, Brunelin J, et al. Perceived ethnic discrimination as a risk factor for psychotic symptoms: a systematic review and meta-analysis. Psychological medicine. 2020;50(7):1077-89. doi: <https://dx.doi.org/10.1017/S003329172000094X>.

2. Chakraborty AT, McKenzie KJ, Hajat S, Stansfeld SA. Racism, mental illness and social support in the UK. Soc Psychiatry Psychiatr Epidemiol. 2010;45(12):1115-24. Epub 20091022. doi: 10.1007/s00127-009-0156-8. PubMed PMID: 19847373.

3. Berg AO, Melle I, Rossberg JI, Romm KL, Larsson S, Lagerberg TV, et al. Perceived discrimination is associated with severity of positive and depression/anxiety symptoms in immigrants with psychosis: a cross-sectional study. BMC psychiatry. 2011;11(1):1-9.

4. Paradies Y, Ben J, Denson N, Elias A, Priest N, Pieterse A, et al. Racism as a determinant of health: a systematic review and meta-analysis. PloS one. 2015;10(9):e0138511.

5. Pearce J, Rafiq S, Simpson J, Varese F. Perceived discrimination and psychosis: a systematic review of the literature. Social psychiatry and psychiatric epidemiology. 2019;54(9):1023-44. doi: <https://dx.doi.org/10.1007/s00127-019-01729-3>.

6. Bécares L, Nazroo J, Stafford M. The buffering effects of ethnic density on experienced racism and health. Health Place. 2009;15(3):670-8. Epub 20081118. doi: 10.1016/j.healthplace.2008.10.008. PubMed PMID: 19117792.

7. Karlsen S, Nazroo JY, McKenzie K, Bhui K, Weich S. Racism, psychosis and common mental disorder among ethnic minority groups in England. Psychol Med. 2005;35(12):1795-803. Epub 20050929. doi: 10.1017/s0033291705005830. PubMed PMID: 16194282.

8. de Freitas DF, Fernandes-Jesus M, Ferreira PD, Coimbra S, Teixeira PM, de Moura A, et al. Psychological correlates of perceived ethnic discrimination in Europe: A meta-analysis. Special Issue: Hate and Violence: Addressing Discrimination Based on Race, Ethnicity, Religion, Sexual Orientation, and Gender Identity. 2018;8(6):712-25. doi: <https://dx.doi.org/10.1037/vio0000215>.

9. Williams DR, Mohammed SA. Discrimination and racial disparities in health: evidence and needed research. Journal of behavioral medicine. 2009;32:20-47.
