# Supplementary material for: The association between racism and psychosis: An umbrella review
Source: PLOS Ment Health. 2025 Sep 24;2(9):e0000401. doi: 10.1371/journal.pmen.0000401 (PMC12798482; doi:10.1371/journal.pmen.0000401)
Supplement: S1 Table — Results of the AMSTAR-2 risk of bias assessment and overview of the total AMSTAR-2 items met by each review. (DOCX) [file pmen.0000401.s005.docx]

## S1 Table. AMSTAR-2 Appraisal.

| **Item** | **Bardol et al., 2020 [**[**1**](#_ENREF_1)**]** | **deFreitas et al., 2018 [**[**2**](#_ENREF_2)**]** | **Paradies et al., 2015 [**[**3**](#_ENREF_3)**]** | **Pearce et al., 2019 [**[**4**](#_ENREF_4)**]** | **Pieterse et al., 2012 [**[**5**](#_ENREF_5)**]** | **Williams and Mohammed, 2009 [**[**6**](#_ENREF_6)**]** | **Williams et al., 2003 [**[**7**](#_ENREF_7)**]** |
| --- | --- | --- | --- | --- | --- | --- | --- |
| 1. Did the research questions and inclusion criteria for the review include the components of PICO? | Yes | No | No | Yes | No | No | No |
| **2. Did the report of the review contain an explicit statement that the review methods were established prior to the conduct of the review and did the report justify any significant deviations from the protocol?** | Partial Yes | No | Partial Yes | No | No | No | No |
| 3. Did the review authors explain their selection of the study designs for inclusion in the review? | Yes | Yes | Yes | Yes | Yes | Yes | Yes |
| **4. Did the review authors use a comprehensive literature search strategy?** | Partial Yes | Partial Yes | Partial Yes | Partial Yes | Partial Yes | No | Partial Yes |
| 5. Did the review authors perform study selection in duplicate? | Yes | Yes | Yes | No | No | No | No |
| 6. Did the review authors perform data extraction in duplicate? | Yes | No | Yes | No | Yes | No | No |
| **7. Did the review authors provide a list of excluded studies and justify the exclusions?** | Yes | No | Partial Yes | Yes | No | No | No |
| 8. Did the review authors describe the included studies in adequate detail? | Partial Yes | No | No | Yes | No | No | No |
| **9a. Did the review authors use a satisfactory technique for assessing the risk of bias (RoB) in individual studies that were included in the review? - RCTs** | N/A | N/A | N/A | N/A | N/A | N/A | N/A |
| **9b. Did the review authors use a satisfactory technique for assessing the risk of bias (RoB) in individual studies that were included in the review? - NRSI** | No | No | No | Yes | No | No | No |
| 10. Did the review authors report on the sources of funding for the studies included in the review? | No | No | No | No | No | No | No |
| **11a. If meta-analysis was performed did the review authors use appropriate methods for statistical combination of results? - RCTs** | N/A | N/A | N/A | N/A | N/A | N/A | N/A |
| **11b. If meta-analysis was performed did the review authors use appropriate methods for statistical combination of results? - NRSI** | Yes | Yes | No | N/A | No | N/A | N/A |
| 12. If meta-analysis was performed, did the review authors assess the potential impact of RoB in individual studies on the results of the meta-analysis or other evidence synthesis? | No | No | Yes | N/A | No | N/A | N/A |
| **13. Did the review authors account for RoB in individual studies when interpreting/discussing the results of the review?** | Yes | No | Yes | Yes | No | No | No |
| 14. Did the review authors provide a satisfactory explanation for, and discussion of, any heterogeneity observed in the results of the review? | Yes | Yes | No | No | Yes | No | No |
| **15. If they performed quantitative synthesis did the review authors carry out an adequate investigation of publication bias (small study bias) and discuss its likely impact on the results of the review?** | Yes | Yes | Yes | N/A | Yes | N/A | N/A |
| 16. Did the review authors report any potential sources of conflict of interest, including any funding they received for conducting the review? | No | No | Yes | Yes | No | No | No |
|  |  |  |  |  |  |  |  |
| **Number of critical item non-endorsement** | **1** | **4** | **2** | **1** | **5** | **5** | **4** |
| **OVERALL RATING** | **LOW** | **CRITICALLY LOW** | **CRITICALLY LOW** | **LOW** | **CRITICALLY LOW** | **CRITICALLY LOW** | **CRITICALLY LOW** |
| **Total items met** | **12 (75%)** | **6 (38%)** | **10 (63%)** | **8 (62%)^1^** | **5 (31%)** | **1 (8%)^1^** | **2 (15%)^1^** |

Results of the AMSTAR-2 risk of bias assessment and overview of the number of AMSTAR-2 items met by each review.

The AMSTAR-2 critical domains (items 2, 4, 7, 9, 11, 13 and 15) are in bold above. The overall quality rating was calculated in line with the AMSTAR-2 guidance: High - No or one non-critical weakness; Moderate - More than one non-critical weakness; Low - One critical flaw with or without non-critical weaknesses; Critically low - More than one critical flaw with or without non-critical weaknesses. Number of critical flaws (number of critical items not met by the review) is also given.

Overview of the number of AMSTAR-2 items met by each review was calculated as the number of all relevant AMSTAR-2 items for which the review was assessed as meeting (either ‘yes’ or ‘partial yes’).

^1^Items related to meta-analytic methods were not relevant to reviews without meta-analyses. The totals for these reviews were instead calculated as a percentage of the total 13 relevant items.

NRSI = non-randomised studies of interventions; PICO = four different potential components of a health question: patient/population/problem, intervention, comparison and outcome; RCTs = randomised controlled trials; ROB = risk of bias.

References

1. Bardol O, Grot S, Oh H, Poulet E, Zeroug-Vial H, Brunelin J, et al. Perceived ethnic discrimination as a risk factor for psychotic symptoms: a systematic review and meta-analysis. Psychological medicine. 2020;50(7):1077-89. doi: <https://dx.doi.org/10.1017/S003329172000094X>.

2. de Freitas DF, Fernandes-Jesus M, Ferreira PD, Coimbra S, Teixeira PM, de Moura A, et al. Psychological correlates of perceived ethnic discrimination in Europe: A meta-analysis. Special Issue: Hate and Violence: Addressing Discrimination Based on Race, Ethnicity, Religion, Sexual Orientation, and Gender Identity. 2018;8(6):712-25. doi: <https://dx.doi.org/10.1037/vio0000215>.

3. Paradies Y, Ben J, Denson N, Elias A, Priest N, Pieterse A, et al. Racism as a determinant of health: a systematic review and meta-analysis. PloS one. 2015;10(9):e0138511.

4. Pearce J, Rafiq S, Simpson J, Varese F. Perceived discrimination and psychosis: a systematic review of the literature. Social psychiatry and psychiatric epidemiology. 2019;54(9):1023-44. doi: <https://dx.doi.org/10.1007/s00127-019-01729-3>.

5. Pieterse AL, Todd NR, Neville HA, Carter RT. Perceived racism and mental health among black american adults: A meta-analytic review. Journal of counseling psychology. 2012;59(1):1-9. doi: 10.1037/a0026208.

6. Williams DR, Mohammed SA. Discrimination and racial disparities in health: evidence and needed research. Journal of behavioral medicine. 2009;32:20-47.

7. Williams DR, Neighbors HW, Jackson JS. Racial/ethnic discrimination and health: findings from community studies. Am J Public Health. 2003;93(2):200-8. doi: 10.2105/ajph.93.2.200. PubMed PMID: 12554570; PubMed Central PMCID: PMCPMC1447717.
