# Supplementary material for: The association between racism and psychosis: An umbrella review
Source: PLOS Ment Health. 2025 Sep 24;2(9):e0000401. doi: 10.1371/journal.pmen.0000401 (PMC12798482; doi:10.1371/journal.pmen.0000401)
Supplement: S2 Table — Summary of risk of bias assessments and scores of the included primary studies, as conducted and reported by the reviews within which they were reported. (DOCX) [file pmen.0000401.s006.docx]

## S2 Table. Primary Studies Risk of Bias.

| **Primary study** | **Review(s) reporting on primary study** | **Risk of bias tool used** | **Risk of bias score** |
| --- | --- | --- | --- |
| Anglin et al., 2014[[1](#_ENREF_1)] | Bardol et al., 2020[[2](#_ENREF_2)] | STROBE  (a score of 1 (presence) or 0 (absence) was coded for each item (total score on 32)) | 23 |
|  | Pearce et al., 2019[[3](#_ENREF_3)] | EPHPP | Selection bias: Weak  Study design: Weak  Confounders: Moderate  Blinding: Moderate  Data collection: Strong  Withdrawals and dropouts: NA given the cross-sectional nature of studies |
| Anglin et al., 2016[[4](#_ENREF_4)] | Bardol et al., 2020[[2](#_ENREF_2)] | STROBE  (a score of 1 (presence) or 0 (absence) was coded for each item (total score on 32)) | 23 |
|  | Pearce et al., 2019[[3](#_ENREF_3)] | EPHPP | Selection bias: Weak  Study design: Weak  Confounders: Weak  Blinding: Moderate  Data collection: Strong  Withdrawals and dropouts: NA given the cross-sectional nature of studies |
| Anglin et al., 2018[[5](#_ENREF_5)] | Bardol et al., 2020[[2](#_ENREF_2)] | STROBE  (a score of 1 (presence) or 0 (absence) was coded for each item (total score on 32)) | 22 |
|  | Pearce et al., 2019[[3](#_ENREF_3)] | EPHPP | Selection bias: Weak  Study design: Weak  Confounders: Weak  Blinding: Moderate  Data collection: Strong  Withdrawals and dropouts: NA given the cross-sectional nature of studies |
| Bécares et al., 2009[[6](#_ENREF_6)] | Bardol et al., 2020[[2](#_ENREF_2)] | STROBE  (a score of 1 (presence) or 0 (absence) was coded for each item (total score on 32)) | 22 |
|  | Paradies et al., 2015[[7](#_ENREF_7)] | Review reported that an assessment was conducted using the author’s own measure | Review reported that an assessment was conducted but did not report the score |
|  | Pearce et al., 2019[[3](#_ENREF_3)] | EPHPP | Selection bias: Moderate  Study design: Weak  Confounders: Strong  Blinding: Moderate  Data collection: Moderate  Withdrawals and dropouts: NA given the cross-sectional nature of studies |
| Berg et al., 2011[[8](#_ENREF_8)] | Bardol et al., 2020[[2](#_ENREF_2)] | STROBE  (a score of 1 (presence) or 0 (absence) was coded for each item (total score on 32)) | 27 |
|  | Paradies et al., 2015[[7](#_ENREF_7)] | Review reported that an assessment was conducted using the author’s own measure | Review reported that an assessment was conducted but did not report the score |
|  | Pearce et al., 2019[[3](#_ENREF_3)] | EPHPP | Selection bias: Moderate  Study design: Weak  Confounders: Weak  Blinding: Moderate  Data collection: Strong  Withdrawals and dropouts: NA given the cross-sectional nature of studies |
| Bowen-Reid and Harrell, 2002[[9](#_ENREF_9)] | Paradies et al., 2015[[7](#_ENREF_7)] | Review reported that an assessment was conducted using the author’s own measure | Review reported that an assessment was conducted but did not report the score |
| Chakraborty et al., 2010[[10](#_ENREF_10)] | Bardol et al., 2020[[2](#_ENREF_2)] | STROBE  (a score of 1 (presence) or 0 (absence) was coded for each item (total score on 32)) | 25 |
|  | Paradies et al., 2015[[7](#_ENREF_7)] | Review reported that an assessment was conducted using the author’s own measure | Review reported that an assessment was conducted but did not report the score |
|  | Pearce et al., 2019[[3](#_ENREF_3)] | EPHPP | Selection bias: Moderate  Study design: Weak  Confounders: Strong  Blinding: Moderate  Data collection: Moderate  Withdrawals and dropouts: NA given the cross-sectional nature of studies |
| Combs et al., 2006[[11](#_ENREF_11)] | Bardol et al., 2020[[2](#_ENREF_2)] | STROBE  (a score of 1 (presence) or 0 (absence) was coded for each item (total score on 32)) | 24 |
|  | Paradies et al., 2015[[7](#_ENREF_7)] | Review reported that an assessment was conducted using the author’s own measure | Review reported that an assessment was conducted but did not report the score |
|  | Pearce et al., 2019[[3](#_ENREF_3)] | EPHPP | Selection bias: Weak  Study design: Weak  Confounders: Weak  Blinding: Moderate  Data collection: Strong  Withdrawals and dropouts: NA given the cross-sectional nature of studies |
| Das-Munshi et al., 2012[[12](#_ENREF_12)] | Pearce et al., 2019[[3](#_ENREF_3)] | EPHPP | Selection bias: Moderate  Study design: Weak  Confounders: Strong  Blinding: Moderate  Data collection: Moderate  Withdrawals and dropouts: NA given the cross-sectional nature of studies |
| El Bouhaddani et al., 2019[[13](#_ENREF_13)] | Bardol et al., 2020[[2](#_ENREF_2)] | STROBE  (a score of 1 (presence) or 0 (absence) was coded for each item (total score on 32)) | 26 |
| Gilvarry et al., 1999[[14](#_ENREF_14)] | Pearce et al., 2019[[3](#_ENREF_3)] | EPHPP | Selection bias: Weak  Study design: Moderate  Confounders: Weak  Blinding: Moderate  Data collection: Moderate  Withdrawals and dropouts: Strong |
| Karlsen and Nazroo, 2002[[15](#_ENREF_15)] | Pearce et al., 2019[[3](#_ENREF_3)] | EPHPP | Selection bias: Moderate  Study design: Weak  Confounders: Weak  Blinding: Moderate  Data collection: Moderate  Withdrawals and dropouts: NA given the cross-sectional nature of studies |
|  | Williams et al., 2003[[16](#_ENREF_16)] | No assessment reported by the review | - |
| Karlsen et al., 2005[[17](#_ENREF_17)] | Bardol et al., 2020[[2](#_ENREF_2)] | STROBE  (a score of 1 (presence) or 0 (absence) was coded for each item (total score on 32)) | 22 |
|  | deFreitas et al., 2018[[18](#_ENREF_18)] | Study quality assessed on Likert-type scale of 0 to 6 and based on the author’s assessment of the method of each research, including the type of sampling, reliability of the instruments, and overall quality of the article | 3 |
|  | Pearce et al., 2019[[3](#_ENREF_3)] | EPHPP | Selection bias: Moderate  Study design: Weak  Confounders: Moderate  Blinding: Moderate  Data collection: Moderate  Withdrawals and dropouts: NA given the cross-sectional nature of studies |
|  | Williams and Mohammed, 2009[[19](#_ENREF_19)] | No assessment reported by the review | - |
| Kong, 2016 (Study 1)[[20](#_ENREF_20)] | Bardol et al., 2020[[2](#_ENREF_2)] | STROBE  (a score of 1 (presence) or 0 (absence) was coded for each item (total score on 32)) | 22 |
|  | Pearce et al., 2019[[3](#_ENREF_3)] | EPHPP | Selection bias: Weak  Study design: Moderate  Confounders: Weak  Blinding: Moderate  Data collection: Strong  Withdrawals and dropouts: Strong |
| Kong, 2016 (Study 2)[[20](#_ENREF_20)] | Bardol et al., 2020[[2](#_ENREF_2)] | STROBE  (a score of 1 (presence) or 0 (absence) was coded for each item (total score on 32)) | 22 |
|  | Pearce et al., 2019[[3](#_ENREF_3)] | EPHPP | Selection bias: Weak  Study design: Moderate  Confounders: Weak  Blinding: Moderate  Data collection: Strong  Withdrawals and dropouts: Strong |
| Oh et al., 2014[[21](#_ENREF_21)] | Bardol et al., 2020[[2](#_ENREF_2)] | STROBE  (a score of 1 (presence) or 0 (absence) was coded for each item (total score on 32)) | 25 |
| Oh et al., 2016[[22](#_ENREF_22)] | Bardol et al., 2020[[2](#_ENREF_2)] | STROBE  (a score of 1 (presence) or 0 (absence) was coded for each item (total score on 32)) | 24 |
|  | Pearce et al., 2019[[3](#_ENREF_3)] | EPHPP | Selection bias: Moderate  Study design: Weak  Confounders: Strong  Blinding: Moderate  Data collection: Strong  Withdrawals and dropouts: NA given the cross-sectional nature of studies |
| Shaikh et al., 2016[[23](#_ENREF_23)] | Bardol et al., 2020[[2](#_ENREF_2)] | STROBE  (a score of 1 (presence) or 0 (absence) was coded for each item (total score on 32)) | 25 |
|  | Pearce et al., 2019[[3](#_ENREF_3)] | EPHPP | Selection bias: Weak  Study design: Weak  Confounders: Moderate  Blinding: Moderate  Data collection: Strong  Withdrawals and dropouts: NA given the cross-sectional nature of studies |
| van de Beek et al., 2017[[24](#_ENREF_24)] | Bardol et al., 2020[[2](#_ENREF_2)] | STROBE  (a score of 1 (presence) or 0 (absence) was coded for each item (total score on 32)) | 24 |
| van der Stelt et al., 2013[[25](#_ENREF_25)] | deFreitas et al., 2018[[18](#_ENREF_18)] | Study quality assessed on Likert-type scale of 0 to 6 and based on the author’s assessment of the method of each research, including the type of sampling, reliability of the instruments, and overall quality of the article | 4 |
| Veling et al., 2007[[26](#_ENREF_26)] | Bardol et al., 2020[[2](#_ENREF_2)] | STROBE  (a score of 1 (presence) or 0 (absence) was coded for each item (total score on 32)) | 25 |
|  | Williams and Mohammed, 2009[[19](#_ENREF_19)] | No assessment reported by the review | - |
| Veling et al., 2008[[27](#_ENREF_27)] | Bardol et al., 2020[[2](#_ENREF_2)] | STROBE  (a score of 1 (presence) or 0 (absence) was coded for each item (total score on 32)) | 27 |
|  | Pearce et al., 2019[[3](#_ENREF_3)] | EPHPP | Selection bias: Moderate  Study design: Moderate  Confounders: Strong  Blinding: Moderate  Data collection: Strong  Withdrawals and dropouts: NA given the cross-sectional nature of studies |
| Vremaroiu and Fodoreanu, 2013[[28](#_ENREF_28)] | Bardol et al., 2020[[2](#_ENREF_2)] | STROBE  (a score of 1 (presence) or 0 (absence) was coded for each item (total score on 32)) | 16 |
|  | Paradies et al., 2015[[7](#_ENREF_7)] | Review reported that an assessment was conducted using the author’s own measure | Review reported that an assessment was conducted but did not report the score |

Summary of risk of bias assessments and scores of the included primary studies, as conducted and reported by the reviews within which they were reported.

Four of the reviews conducted risk of bias assessments; however, one review [[7](#_ENREF_7)] did not report the tool used or the assessment scores for the primary studies. The remaining three reviews did not report conducting a risk of bias assessment on the included primary studies. Therefore, risk of bias assessments are only reported below for three reviews.

Note: several papers are cited differently between reviews. As such, Anglin [[4](#_ENREF_4)] is reported as Anglin et al., 2014b [ref 11] in Pearce et al. [[3](#_ENREF_3)]; Anglin [[5](#_ENREF_5)] is reported as Anglin et al., 2016 [ref 39] in Pearce et al. [[3](#_ENREF_3)]; and Oh [[22](#_ENREF_22)] is reported as Oh, 2016 within the primary study characteristics tables in Bardol et al. [[2](#_ENREF_2)]. Additionally, Kong [[20](#_ENREF_20)] is shown twice as the paper includes two relevant studies.

The overall STROBE score quality are reported by Bardol et al. [[2](#_ENREF_2)] as: very good quality (26–32); good quality (20–26); average quality (14–20); and poor quality (<14).

References

1. Anglin DM, Lighty Q, Greenspoon M, Ellman LM. Racial discrimination is associated with distressing subthreshold positive psychotic symptoms among US urban ethnic minority young adults. Social psychiatry and psychiatric epidemiology. 2014;49:1545-55.

2. Bardol O, Grot S, Oh H, Poulet E, Zeroug-Vial H, Brunelin J, et al. Perceived ethnic discrimination as a risk factor for psychotic symptoms: a systematic review and meta-analysis. Psychological medicine. 2020;50(7):1077-89. doi: <https://dx.doi.org/10.1017/S003329172000094X>.

3. Pearce J, Rafiq S, Simpson J, Varese F. Perceived discrimination and psychosis: a systematic review of the literature. Social psychiatry and psychiatric epidemiology. 2019;54(9):1023-44. doi: <https://dx.doi.org/10.1007/s00127-019-01729-3>.

4. Anglin DM, Greenspoon M, Lighty Q, Ellman LM. Race-based rejection sensitivity partially accounts for the relationship between racial discrimination and distressing attenuated positive psychotic symptoms. Early Interv Psychiatry. 2016;10(5):411-8. Epub 20140918. doi: 10.1111/eip.12184. PubMed PMID: 25234291.

5. Anglin DM, Lui F, Espinosa A, Tikhonov A, Ellman L. Ethnic identity, racial discrimination and attenuated psychotic symptoms in an urban population of emerging adults. Early Interv Psychiatry. 2018;12(3):380-90. Epub 20160128. doi: 10.1111/eip.12314. PubMed PMID: 26818635.

6. Bécares L, Nazroo J, Stafford M. The buffering effects of ethnic density on experienced racism and health. Health Place. 2009;15(3):670-8. Epub 20081118. doi: 10.1016/j.healthplace.2008.10.008. PubMed PMID: 19117792.

7. Paradies Y, Ben J, Denson N, Elias A, Priest N, Pieterse A, et al. Racism as a determinant of health: a systematic review and meta-analysis. PloS one. 2015;10(9):e0138511.

8. Berg AO, Melle I, Rossberg JI, Romm KL, Larsson S, Lagerberg TV, et al. Perceived discrimination is associated with severity of positive and depression/anxiety symptoms in immigrants with psychosis: a cross-sectional study. BMC psychiatry. 2011;11(1):1-9.

9. Bowen-Reid TL, Harrell JP. Racist Experiences and Health Outcomes: An Examination of Spirituality as a Buffer. Journal of Black Psychology. 2002;28(1):18-36. doi: 10.1177/0095798402028001002.

10. Chakraborty AT, McKenzie KJ, Hajat S, Stansfeld SA. Racism, mental illness and social support in the UK. Soc Psychiatry Psychiatr Epidemiol. 2010;45(12):1115-24. Epub 20091022. doi: 10.1007/s00127-009-0156-8. PubMed PMID: 19847373.

11. Combs DR, Penn DL, Cassisi J, Michael C, Wood T, Wanner J, et al. Perceived Racism as a Predictor of Paranoia Among African Americans. Journal of Black Psychology. 2006;32(1):87-104. doi: 10.1177/0095798405283175.

12. Das-Munshi J, Bécares L, Boydell JE, Dewey ME, Morgan C, Stansfeld SA, et al. Ethnic density as a buffer for psychotic experiences: findings from a national survey (EMPIRIC). Br J Psychiatry. 2012;201(4):282-90. Epub 20120726. doi: 10.1192/bjp.bp.111.102376. PubMed PMID: 22844021; PubMed Central PMCID: PMCPMC3461446.

13. El Bouhaddani S, van Domburgh L, Schaefer B, Doreleijers TAH, Veling W. Psychotic experiences among ethnic majority and minority adolescents and the role of discrimination and ethnic identity. Soc Psychiatry Psychiatr Epidemiol. 2019;54(3):343-53. Epub 20190114. doi: 10.1007/s00127-019-01658-1. PubMed PMID: 30643926.

14. Gilvarry CM, Walsh E, Samele C, Hutchinson G, Mallett R, Rabe-Hesketh S, et al. Life events, ethnicity and perceptions of discrimination in patients with severe mental illness. Soc Psychiatry Psychiatr Epidemiol. 1999;34(11):600-8. doi: 10.1007/s001270050181. PubMed PMID: 10651179.

15. Karlsen S, Nazroo JY. Relation between racial discrimination, social class, and health among ethnic minority groups. Am J Public Health. 2002;92(4):624-31. doi: 10.2105/ajph.92.4.624. PubMed PMID: 11919063; PubMed Central PMCID: PMCPMC1447128.

16. Williams DR, Neighbors HW, Jackson JS. Racial/ethnic discrimination and health: findings from community studies. Am J Public Health. 2003;93(2):200-8. doi: 10.2105/ajph.93.2.200. PubMed PMID: 12554570; PubMed Central PMCID: PMCPMC1447717.

17. Karlsen S, Nazroo JY, McKenzie K, Bhui K, Weich S. Racism, psychosis and common mental disorder among ethnic minority groups in England. Psychol Med. 2005;35(12):1795-803. Epub 20050929. doi: 10.1017/s0033291705005830. PubMed PMID: 16194282.

18. de Freitas DF, Fernandes-Jesus M, Ferreira PD, Coimbra S, Teixeira PM, de Moura A, et al. Psychological correlates of perceived ethnic discrimination in Europe: A meta-analysis. Special Issue: Hate and Violence: Addressing Discrimination Based on Race, Ethnicity, Religion, Sexual Orientation, and Gender Identity. 2018;8(6):712-25. doi: <https://dx.doi.org/10.1037/vio0000215>.

19. Williams DR, Mohammed SA. Discrimination and racial disparities in health: evidence and needed research. Journal of behavioral medicine. 2009;32:20-47.

20. Kong DT. Ethnic minorities' paranoia and self-preservative work behaviors in response to perceived ethnic discrimination, with collective self-esteem as a buffer. J Occup Health Psychol. 2016;21(3):334-51. Epub 20151214. doi: 10.1037/ocp0000013. PubMed PMID: 26652266.

21. Oh H, Yang LH, Anglin DM, DeVylder JE. Perceived discrimination and psychotic experiences across multiple ethnic groups in the United States. Schizophrenia research. 2014;157(1-3):259-65.

22. Oh H, Cogburn CD, Anglin D, Lukens E, DeVylder J. Major discriminatory events and risk for psychotic experiences among Black Americans. Am J Orthopsychiatry. 2016;86(3):277-85. Epub 20160310. doi: 10.1037/ort0000158. PubMed PMID: 26963179.

23. Shaikh M, Ellett L, Dutt A, Day F, Laing J, Kroll J, et al. Perceived ethnic discrimination and persecutory paranoia in individuals at ultra-high risk for psychosis. Psychiatry Res. 2016;241:309-14. Epub 20160512. doi: 10.1016/j.psychres.2016.05.006. PubMed PMID: 27232552.

24. van de Beek MH, van der Krieke L, Schoevers RA, Veling W. Social exclusion and psychopathology in an online cohort of Moroccan-Dutch migrants: Results of the MEDINA-study. PLoS One. 2017;12(7). doi: <https://doi.org/10.1371/journal.pone.0179827>. PubMed PMID: 1917696424.

25. van der Stelt O, Boubakri D, Feltzer M. Migration Status, Familial Risk for Mental Disorder, and Schizotypal Personality Traits. Europe’s Journal of Psychology. 2013;9(3):552-71. doi: 10.5964/ejop.v9i3.623.

26. Veling W, Selten J-P, Susser E, Laan W, Mackenbach JP, Hoek HW. Discrimination and the incidence of psychotic disorders among ethnic minorities in The Netherlands. International journal of epidemiology. 2007;36(4):761-8.

27. Veling W, Hoek HW, Mackenbach JP. Perceived discrimination and the risk of schizophrenia in ethnic minorities: a case-control study. Social psychiatry and psychiatric epidemiology. 2008;43:953-9.

28. Vremaroiu A-M, Fodoreanu L. Perceived discrimination, a risk factor for developing psychoses in Romanian emigrants? Clujul medical (1957). 2013;86(1):53-6.
