# Supplementary material for: The association between racism and psychosis: An umbrella review
Source: PLOS Ment Health. 2025 Sep 24;2(9):e0000401. doi: 10.1371/journal.pmen.0000401 (PMC12798482; doi:10.1371/journal.pmen.0000401)
Supplement: Attachment — Submitted filename: Response to Reviewers.pdf [file pmen.0000401.s007.pdf]

DIVISION OF PSYCHIATRY

India Francis-Crossley
Division of Psychiatry, 6th floor Maple House
149 Tottenham Court Rd
London
W1T 7NF
21st May 2025

Juan Felipe Cardona, Ph.D.
Academic Editor
PLOS Mental Health

Dear Dr Juan Felipe Cardona and Reviewers,

Re: The association between racism and psychosis: An umbrella review – Response to Reviewers

Thank you very much for your time reviewing this paper, we really appreciate your rigorous and positive review of our work. We note that you highlight both the strengths of the paper as well as some limitations which we discussed within the manuscript, as well as some feedback for suggested amendments to the review.

Please find responses to your review comments below.

Yours sincerely,

India Francis-Crossley, Professor James B. Kirkbride and co-authors

---

Reviewer #1: Introduction:

• Line 69 could benefit from further explaining the specific adverse outcomes of people with psychosis

We have amended this section to ascribe reference 5 to the relevant information "…and face barriers to receiving physical healthcare.", as well as provide some additional explanation of adverse outcomes. This section now reads as follows (**lines 67–70**):

"People living with psychosis also experience high rates of unemployment [3], barriers to accessing physical healthcare [5], and continue to be exposed to elevated levels of stigma and social exclusion [6]. These factors can further extend their health and social inequalities."

• Some inconsistencies in the way statistics are reported (numeric vs spelled out)

We have updated the reporting to ensure it is consistently numeric:

"A recent meta-analysis conducted in the US also showed that people from Black ethnic backgrounds were two times as likely to be diagnosed with a psychotic disorder than White individual [9], with another estimate

suggesting that people from Latine and Black ethnicities were **2 and 4** times more likely to experience psychosis, respectively, than people from White backgrounds [11].” (**lines 79–80**)

• Lines 95 – 103 are not especially clear in the flow of explaining the differing levels of racism. There could be edits for overall logical flow.
Thank you for this feedback, we have edited this section and created a new paragraph to increase clarity of the explanation. This section now reads as follows (**lines 97–109**):

“Many of these factors may be broadly conceptualised under the umbrella term, systemic racism – one of many forms of racism.

Racism itself can be broadly defined as the unequal distribution of power, resources and/or opportunities due to a person’s ethnicity or race. The act or experience of racism is defined as racial/ethnic discrimination and include behaviours such as exclusion, attacks and microaggressions. Racism can take many different forms, including interpersonal, internalised, vicarious and structural/institutional racism [20, 24, 25], and can therefore be pervasive in the ways in which it impacts people from racialised backgrounds, from personal through to systemic levels of oppression. In fact, the impact of such racial/ethnic discrimination may also be passed on to later generations as generational trauma through epigenetic factors, such as in the descendants of enslaved peoples [26].”

Methods:
• It would be beneficial to further explain the adaptation in Google Scholar that was used for searching
We have included an overview of the adapted search domains to explain the adaptation, and signposted the reader to supplement 2 which contains full details on the google scholar search methodology for conciseness of the manuscript. The update is as follows (**lines 147–150**):

“Additionally, we searched Google Scholar using an adapted version of the search concepts, using the following search terms: 1) racism/racial discrimination, 2) psychosis/schizophrenia, and 3) systematic review/meta-analysis (further details in Supplement 2).”

Overall:
• Highlighting the most correlated causal results could make the results and discussion stronger. Authors could consider a figure to highlight the relationships.
We have included an overview of the most correlated causal results. On reflection, we decided to include this narratively rather than within a figure due to the heterogeneity in the reporting of the data and effect sizes. Therefore, we have made the following addition (**lines 637–644**):

“The largest effect sizes reported (correlation coefficients and odds ratios, respectively) were observed in non-clinical settings between perceived racial/ethnic discrimination and schizotypy in participants with a family history of psychopathology (r=0.51; derived p=0.01) [68] and between physical racial attacks and psychosis symptoms (OR 4.77, 95% CI 2.32, 9.80) [29]. Several other studies also observed large odds ratios of at least 2.00 [56, 61, 63]. The largest effect size reported in the clinical setting was observed between perceived racial/ethnic discrimination and prodromal psychotic symptoms in UHR participants (r=0.33, p=0.009) [66].”

Reviewer #2: Peer Review of "The Association Between Racism and Psychosis: An Umbrella Review"
Methodology
The authors conducted a comprehensive umbrella review, systematically searching multiple databases (Medline, Embase, PsycINFO, ProQuest Central, and Google Scholar) for systematic reviews and meta-analyses related to racial/ethnic discrimination and psychosis. The methodology is well-structured, adhering to PRISMA guidelines

and pre-registering the protocol (CRD42023400656).

Key methodological strengths include:

Rigorous Inclusion Criteria: The review included systematic reviews and meta-analyses that specifically investigated non-organic psychosis and racial/ethnic discrimination, ensuring a focused analysis.
Risk of Bias Assessment: The authors employed the AMSTAR-2 tool to assess the quality of included reviews, which is a recognized method for evaluating systematic reviews.
Data Extraction and Synthesis: Data were extracted narratively, and the authors attempted meta-analyses where possible, although they faced challenges due to heterogeneity among studies.
However, there are some limitations:

High Heterogeneity: The review noted significant heterogeneity in the studies, which complicates the synthesis of results and limits the generalizability of findings.
Quality of Included Reviews: Most reviews were rated as low or critically low quality, which raises concerns about the reliability of the conclusions drawn from the synthesized evidence.
Clarity
The manuscript is generally well-written and organized, with a clear structure that guides the reader through the introduction, methodology, results, and discussion. The use of headings and subheadings enhances readability.

Strengths in clarity include:

Clear Definitions: The authors provide clear definitions of key terms, such as "racial/ethnic discrimination" and "psychosis," which helps contextualize the research.
Logical Flow: The progression from the introduction to the results and discussion is logical, making it easy for readers to follow the authors' arguments.
Areas for improvement:

Complexity of Language: Some sections could benefit from simpler language or clearer explanations, particularly when discussing statistical analyses and methodological terms.
We have simplified language and provide clearer explanations of statistical and methodological concepts throughout the review as follows:

Lines 150–153: “We also conducted forward- and backward-citation searching (searching for reviews included in the systematic review reference lists, and reviews that cited the included systematic reviews) of included systematic reviews to identify any potential reviews missed by our initial searches.”

Lines 227–229: “As AMSTAR-2 has been criticised for low scoring when applied to observational studies [42], we also calculated the number of items each review met (completely or partially) to provide further detail.”

Lines 232–235: “We planned to perform meta-analyses where there were commonalities in how the effect sizes between psychosis and racial/ethnic discrimination were measured in at least five primary studies, and where both the effect size and a measure of standard error were reported or could be estimated.”

Lines 447–450: “Furthermore, five of the six relevant primary studies [56, 57, 59, 60, 69] from one of these reviews [49] were also reported by two other included reviews [47, 50] (**Fig 2**) which both did disaggregate data for psychosis and also found evidence of an effect of racial/ethnic discrimination on psychosis.”

Lines 749–752: "Longitudinal study designs may help us to elucidate whether there are specific times in the life course where experiences of racial/ethnic discrimination are most impactful, as well as to explore the mechanisms underlying the association."

Figures and Tables: The inclusion of figures (e.g., PRISMA diagram) and tables summarizing key findings could enhance understanding, but they should be clearly labeled and referenced in the text.
Thank you for this suggestion. We have edited the captions of the included figures and tables to increase clarity as follows:

Line 271: "Fig 1 **–** PRISMA diagram to identify reviews included in this umbrella review"

Line 293: "Table 1 **–** Main characteristics of the reviews included in our umbrella review"

Line 307: "Table 2 **–** Characteristics of the primary studies identified by the reviews included in this umbrella review"

Line 343: "Fig 2 **–** Citation matrix of overlap in reporting of primary studies by the included reviews"

Line 376: "Table 3 **–** Participant characteristics from the primary studies, as reported by reviews included in this umbrella review"

Line 426: "Table 4 – Observed associations and meta-analysis results, as reported by reviews included in this umbrella review"

Results
The results of the umbrella review indicate a consistent positive association between racial/ethnic discrimination and psychosis, with all included reviews finding evidence supporting this relationship. Notably:

Quantitative Findings: The meta-analyses reported significant odds ratios for psychotic symptoms and experiences, suggesting a robust link between discrimination and psychosis.
Population Differences: The authors observed stronger evidence for psychotic outcomes in non-clinical populations compared to clinical samples, which is an important distinction.
However, the results are tempered by:

Quality of Evidence: The overall quality of the evidence is low, with many studies being cross-sectional and suffering from methodological limitations.
Need for Further Research: The authors emphasize the necessity for high-quality longitudinal studies to better understand the causal pathways between racial/ethnic discrimination and psychosis.
Conclusion
Overall, the manuscript presents a valuable synthesis of the existing literature on the relationship between racism and psychosis. While the methodology is sound and the results are significant, the low quality of the included studies and high heterogeneity highlight the need for caution in interpreting the findings. Future research should aim to address these gaps, focusing on high-quality longitudinal studies to clarify the mechanisms at play. The clarity of the writing is generally good, but some sections could be simplified for broader accessibility.
Thank you for this feedback, we have reviewed the manuscript and made changes to simplify the language for greater accessibility. Please see these changes above in response to the 'Complexity of Language' comment.
