# Supplementary material for: The association between racism and psychosis: An umbrella review
Source: PLOS Ment Health. 2025 Sep 24;2(9):e0000401. doi: 10.1371/journal.pmen.0000401 (PMC12798482; doi:10.1371/journal.pmen.0000401)
Supplement: Attachment — Submitted filename: Response_to_Reviewers_auresp_2.pdf [file pmen.0000401.s008.pdf]

India Francis-Crossley
Division of Psychiatry, 6th floor Maple House
149 Tottenham Court Rd
London
W1T 7NF
26th June 2025

Juan Felipe Cardona, Ph.D.
Academic Editor
PLOS Mental Health

Dear Dr Juan Felipe Cardona and Reviewers,

Re: The association between racism and psychosis: An umbrella review – Response to Reviewers

Thank you for your review and second round of comments on our paper. We appreciate your time and feedback on our work and note your positive comments as well as the additional suggestions for improvement.

Please find responses to your review comments below.

Yours sincerely,

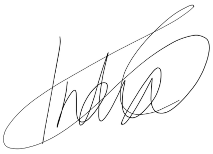

India Francis-Crossley, Professor James B. Kirkbride and co-authors

---

Journal Requirements:
1. As required by our policy on Data Availability, please ensure your manuscript or supplementary information includes the following:

As per your Journal Requirement, specified below, that the following "information can be included in the main text, supplementary information, or relevant data repository", we have decided to host the detailed data requirements below in a data repository; see https://doi.org/10.17605/OSF.IO/GX3DQ

A numbered table of all studies identified in the literature search, including those that were excluded from the analyses. Included – please find these data within the data repository available at the following DOI: https://doi.org/10.17605/OSF.IO/GX3DQ

For every excluded study, the table should list the reason(s) for exclusion. (Please see above link to data repository)

If any of the included studies are unpublished, include a link (URL) to the primary source or detailed information about how the content can be accessed. – N/A

A table of all data extracted from the primary research sources for the systematic review and/or meta-analysis. The table must include the following information for each study: Included – please find these data within the data repository available at the following DOI: https://doi.org/10.17605/OSF.IO/GX3DQ

Name of data extractors and date of data extraction (Please see above link to data repository)

Confirmation that the study was eligible to be included in the review. (Please see above link to data repository)

All data extracted from each study for the reported systematic review and/or meta-analysis that would be needed to replicate your analyses. Included – please find these data within the data repository available at the following DOI: https://doi.org/10.17605/OSF.IO/GX3DQ

If data or supporting information were obtained from another source (e.g. correspondence with the author of the original research article), please provide the source of data and dates on which the data/information were obtained by your research group. Included – please find these data and information within the data repository available at the following DOI: https://doi.org/10.17605/OSF.IO/GX3DQ

If applicable for your analysis, a table showing the completed risk of bias and quality/certainty assessments for each study or outcome. Please ensure this is provided for each domain or parameter assessed. For example, if you used the Cochrane risk-of-bias tool for randomized trials, provide answers to each of the signalling questions for each study. If you used GRADE to assess certainty of evidence, provide judgements about each of the quality of evidence factor. This should be provided for each outcome. Included – please see S1 Table AMSTAR-2 Appraisal (Supplement Tables file).

An explanation of how missing data were handled. – Included as applicable to this review within the methodology (lines 220–226) and results (lines 473–478) sections of the paper.

This information can be included in the main text, supplementary information, or relevant data repository. Please note that providing these underlying data is a requirement for publication in this journal, and if these data are not provided your manuscript might be rejected.

2. Please review your reference list to ensure that it is complete and correct. If you have cited papers that have been retracted, please include the rationale for doing so in the manuscript text, or remove these references and replace them with relevant current references. Any changes to the reference list should be mentioned in the rebuttal letter that accompanies your revised manuscript. If you need to cite a retracted article, indicate the article's retracted status in the References list and also include a citation and full reference for the retraction notice. Complete

Reviewer comments

Reviewer #1:

Thank you for the opportunity to review this important and timely manuscript. This paper provides a much-needed synthesis of existing systematic reviews on a critically understudied area in mental health research. The topic is highly relevant, the methodology is rigorous, and the findings contribute meaningfully to the field. I commend the authors on their transparent reporting, use of a pre-registered protocol, and thorough adherence to PRISMA and Cochrane guidance.

Strengths:

The study addresses a pressing and understudied issue: the role of racial/ethnic discrimination in psychosis.
Methodological rigor is evident in the comprehensive search strategy, dual screening, and use of AMSTAR-2.
The narrative synthesis is clear, well-structured, and appropriately cautious given the heterogeneity and quality of included reviews.
Meta-analytic findings are presented with nuance, and the use of both clinical and non-clinical samples is a particular strength.
Terminological choices (e.g., "racialised backgrounds," "Latine") demonstrate cultural sensitivity and reflexivity.

Suggestions for Minor Revision:
Interpretation of Heterogeneity:
Given the high heterogeneity in several reported meta-analyses (e.g., $I^2 = 79\%$), I recommend including a brief interpretive discussion of how this may affect the robustness or generalizability of the findings.
We have amended the section discussing high heterogeneity to highlight these potential impacts:

'Nonetheless, review quality was low or critically low, sometimes with high levels of heterogeneity in meta-analyses which could impact generalisability and reduce the robustness of findings.' (lines 799–801).

Terminology Clarification:
The authors adopt inclusive and up-to-date terminology throughout (e.g., avoiding BAME). It may be helpful to briefly address how historical terminology in older reviews was handled or interpreted, especially when terminology was inconsistent or outdated.
We have updated our terminology sub-section within the methodology section to include additional explanation for how and why we used the terminology and to improve clarity and which terminology we did and did not change:

'We generally followed the nomenclature used in the original reviews when reporting effects in specific racial/ethnic groups (i.e. Black, African-American, Asian etc.). Exceptions to this included our use of the term "Latine", as a non-gendered term in the place of latino/a/x, and our use of "racialised background" to refer to people who identified with, or were reported as being from a marginalised racial or ethnic group. We used such terminology as an alternative to potential outdated terms such as BAME/BME or "Blacks" as these are exclusionary, can be dehumanising, and focus more on socially-constructed categories rather than the experiences people from racialised backgrounds face. Similarly, when describing experiences of racism, we referred to "racial/ethnic discrimination" to cover the full breadth of experiences reported by the included reviews.' (lines 136–145).

Expanded Implications:
The discussion on public health implications is well-stated, but could benefit from a slightly more detailed outline of potential intervention targets (such as structural vs interpersonal interventions, implications for health services, or culturally grounded early intervention models).
We have expanded the discussion of interventions to include additional intervention targets, as suggested:

'Interventions should also be holistic, both targeting systems that generate racism to address the structural conditions that permit experiences of racism to perpetuate, and to provide support to individuals to help mitigate the negative psychological impacts of racism. An example of a new national intervention from the UK is the Patient and carer race equality (PCREF) framework [87], which has been developed in collaboration with patients, carers and community organisations and introduced across NHS England Trusts. This anti-racism framework aims to provide Trusts with the tools to interrogate and tackle racism within their practices and policies to address the current racial/ethnic inequalities. Interventions could also look to support culturally- and community- grounded early intervention for those experiencing or at risk of experiencing racial/ethnic discrimination, such as the

programmes described and discussed by Jones and Neblett [88]. Since experiences of racial/ethnic discrimination can differ by country and both between and within racialised groups [21, 89-92], such interventions should provide specific support relating to their experiences. Additionally, we need more research investment to understand the most appropriate intersectional interventions to prevent mental ill health in people who face discrimination as a result of multiple intersecting marginalised identities, including by sexuality, gender, and religion [92].' (lines 913–929).

Proofreading:
A few minor typographic and formatting issues (e.g., spacing inconsistencies, capitalization) remain and can be addressed in a final edit.
Thank you for highlighting these issues. We have been through the manuscript carefully to remove these errors, and in the event of acceptance at the Journal, we will work with their copy editor to ensure accuracy of the final, published work.

Reviewer #3:
General comments and some key concerns:
Dear authors, thank you for making the revision and responding to the comments as a way to improve on the manuscript titled "The association between racism and psychosis: An umbrella review". However, the following are some few issues highlighted that need further attention.

1. Methodology

The authors should use "Methodology" instead of "Materials and Methods".
This section had been named "Materials and Methods" in line with the *PLoS Mental Health* manuscript organisation guidelines suggested section name but has been updated to "Methodology" at the reviewer's suggestion (renaming sections is accepted by *PLoS Mental Health*). (line 127).

2. Results

The authors should include the flow chart of how papers included in the study were arrived at.
This information has been included within the PRISMA diagram – please see Fig 1 (line 280, the figure is included as an attachment within the submission).

The authors should present the percentages to 1 decimal place in the whole document and they should be reported as %(n) and not n(%).
Percentages have been rounded to 1 decimal place where they were previously 2, and the order of reporting for n and % has been switched to %(n) where previously reported as n(%) as follows:

Line 189–191: '…following training on the first **30% (n=570)** of the initial title and abstract screening records to ensure high inter-rater reliability (IRR)…'

Line 271–274: 'Our initial search identified 2601 records (Fig 1), of which **26.9% (n=700)** were duplicates. The remaining 1901 records were screened, **86.6% (n=1647)** of which were excluded at title screen (IRR=0.85), **9.9% (n=188)** at abstract screen (IRR=0.83) and **3.1% (n=59)** at full text screen (IRR=0.82).'

In table 2 and 3, the percentages should be removed from each value since it is already included in the sub-title in the column. However, this was not affected from the previous comments to the authors.
Percentage signs have been removed from the relevant columns in table 2 (line 324) and table 3 (line 535).

What is the unit s of age in table 3 and it should be written as age (years or months or weeks depending on the interest)?
The age subtitle has been updated to reference the units (years) and ‘years’ has been removed from the relevant ‘Range’ data in keeping with the above comment (Table 3 – line 535).

The results section needs to be rearranged so that it can flow.
Thank you for your suggestion. We have carefully re-read the results section and discussed this amongst the co-authors. We feel that the results are presented in alignment with the flow of information provided in the introduction and methodology sections, and importantly, follow the analysis and reporting approach we outlined in the methods. We feel that changing this would disrupt the flow of the manuscript and reduce the overall coherence of the piece, though of course we would be happy to work with the editor and/or reviewer if they had specific suggestions for where the flow could be improved. We would like to note that if this comment is relating to the embedded inclusion of tables and figures, this is due to the journal submission guidelines and therefore we are not able to move these to the end of the manuscript for this submission.

3. Conclusion
The authors should include the conclusion based on the findings from the study.
We have added a conclusion section as follows:

‘This umbrella review provides evidence of associations between racial/ethnic discrimination and psychosis in both clinical and non-clinical populations. This may explain some of the excess risk seen in racialised groups across many countries across Europe [28] as well as in the US, Canada and Australia. Despite this, we observed notable issues in the extant literature, including methodological heterogeneity, review quality and a focus on interpersonal racism over other forms including structural and institutional racism. We recommend the need for high quality, longitudinal and co-produced research that explores the mechanisms and interventions that, respectively, could produce and prevent excess psychosis risk that arise after pervasive exposure to multiple, ongoing forms of racism in society.’ (lines 958–967).
