# Supplementary material for: The association between racism and psychosis: An umbrella review
Source: PLOS Ment Health. 2025 Sep 24;2(9):e0000401. doi: 10.1371/journal.pmen.0000401 (PMC12798482; doi:10.1371/journal.pmen.0000401)
Supplement: Attachment — Submitted filename: Response_to_Reviewers_auresp_3.pdf [file pmen.0000401.s009.pdf]

India Francis-Crossley
Division of Psychiatry, 4th floor Maple House
149 Tottenham Court Rd
London
W1T 7NF
16th July 2025

Juan Felipe Cardona, Ph.D.
Academic Editor
*PLOS Mental Health*

Dear Dr Juan Felipe Cardona and Reviewers,

**Re: The association between racism and psychosis: An umbrella review – Response to Reviewers**

Thank you for your review and third round of comments on our paper. We appreciate your positive comments as well as your time and feedback on our work.

Please find responses to your review comments below.

Yours sincerely,

India Francis-Crossley, Professor James B. Kirkbride and co-authors

---

**Editor comments**

Thank you once again for your thoughtful resubmission of the manuscript. I appreciate the sustained effort that you and your co-authors have dedicated to addressing the reviewers' feedback across rounds. Please accept my apologies for the delay in response. The evaluation process was prolonged due to reviewer availability and internal considerations. We appreciate your patience and continued commitment to the process. After a careful assessment of the revised version and based on the comments from Reviewer 1, I am pleased to report that your manuscript is very close to being ready for acceptance. However, we would kindly ask you to consider a small number of minor editorial revisions, which will help ensure clarity and consistency in the final version.

Specifically, expand the concluding paragraph to better summarise the review's key findings and implications for policy and future research, provide a brief clarification on the exclusion of grey literature in your search strategy, and how this might impact the review's comprehensiveness or

introduce potential bias?. Include a concise summary of the types of psychotic outcomes such as clinical diagnosis, subclinical symptoms, considered across included studies to enhance the interpretability of your synthesis.

- **Expand the concluding paragraph to better summarise the review's key findings and implications for policy and future research:**
  - We have updated the conclusions section to provide greater details on the key findings and implications:

    'This umbrella review provides consistent evidence of associations between racial/ethnic discrimination and psychosis in both clinical and non-clinical populations. There was a greater evidence base for the latter with 13 of the 16 primary studies included observing statistically significant positive associations and several of those reporting large odds ratios of at least 2.00 [29, 56, 61, 63]. These findings may help to explain the excess risk seen in racialised groups in many countries across Europe [28] as well as in the US, Canada and Australia.

    Despite this, we observed notable issues in the extant literature, including methodological heterogeneity, review quality and a focus on interpersonal racism over other forms including structural and institutional racism. We recommend the need for high quality, longitudinal research that explores the mechanisms and interventions that, respectively, could produce and prevent excess psychosis risk that arise after pervasive exposure to multiple, ongoing forms of racism in society. The findings of this umbrella review point towards a role for holistic approaches to clinical practice which include targeted interventions for those who may experience racial/ethnic discrimination, education for clinical staff on the impact of racism and how to avoid re-traumatising those receiving care, as well as interventions that target the systems that uphold and enable racism. Future research should also aim to identify protective factors which can be integrated into clinical interventions and policy to improve people's mental wellbeing and tackle racism.' (lines 808–830)

- **Provide a brief clarification on the exclusion of grey literature in your search strategy, and how this might impact the review's comprehensiveness or introduce potential bias**:
  - We have included mention of 'the grey literature' within the methodology section to clarify its exclusion (line 180)
  - We have also discussed the potential impact of excluding grey literature within the limitations in the discussion section:

    'While there is a small possibility we missed unpublished reviews, reviews only indexed in Google Scholar, or those in the grey literature, our backward- and forward- citation searches would have been very likely to alert us to any relevant extant reviews we had missed in our original search; this process led to the identification of two published reviews we included in this umbrella review, but no relevant grey literature, suggesting the impact of omitting the grey literature in our study design would have been small.'

(lines 686–691)

- **Include a concise summary of the types of psychotic outcomes such as clinical diagnosis, subclinical symptoms, considered across included studies to enhance the interpretability of your synthesis**
    - We extended the discussion of psychotic outcomes within the inclusion criteria section to include an explanation to improve clarity:

      'For the purpose of this review we defined psychotic experiences and symptoms as disturbances in perception, thought or belief, such as delusions and hallucinations, and differentiated between reviews and original studies that reported these in non-clinical (i.e. population-based) and clinical (i.e. help-seeking) samples.'
      (lines 173–177)

These refinements are intended to reinforce the clarity and impact of your manuscript.

Importantly, no further responses are needed to Reviewer 2, and we ask you to disregard those comments at this stage.

**Reviewer comments**
None
